# Sustainable Management in Crop Monocultures: The Impact of Retaining Forest on Oil Palm Yield

**DOI:** 10.1371/journal.pone.0091695

**Published:** 2014-03-17

**Authors:** Felicity A. Edwards, David P. Edwards, Sean Sloan, Keith C. Hamer

**Affiliations:** 1 School of Biology, University of Leeds, Leeds, West Yorkshire, United Kingdom; 2 Centre for Tropical Environmental and Sustainability Science (TESS) and School of Marine and Tropical Biology, James Cook University, Cairns, Queensland, Australia; 3 Department of Animal and Plant Sciences, University of Sheffield, Sheffield, South Yorkshire, United Kingdom; Instituto de Higiene e Medicina Tropical, Portugal

## Abstract

Tropical agriculture is expanding rapidly at the expense of forest, driving a global extinction crisis. How to create agricultural landscapes that minimise the clearance of forest and maximise sustainability is thus a key issue. One possibility is protecting natural forest within or adjacent to crop monocultures to harness important ecosystem services provided by biodiversity spill-over that may facilitate production. Yet this contrasts with the conflicting potential that the retention of forest exports dis-services, such as agricultural pests. We focus on oil palm and obtained yields from 499 plantation parcels spanning a total of ≈23,000 ha of oil palm plantation in Sabah, Malaysian Borneo. We investigate the relationship between the extent and proximity of both contiguous and fragmented dipterocarp forest cover and oil palm yield, controlling for variation in oil palm age and for environmental heterogeneity by incorporating proximity to non-native forestry plantations, other oil palm plantations, and large rivers, elevation and soil type in our models. The extent of forest cover and proximity to dipterocarp forest were not significant predictors of oil palm yield. Similarly, proximity to large rivers and other oil palm plantations, as well as soil type had no significant effect. Instead, lower elevation and closer proximity to forestry plantations had significant positive impacts on oil palm yield. These findings suggest that if dipterocarp forests are exporting ecosystem service benefits or ecosystem dis-services, that the net effect on yield is neutral. There is thus no evidence to support arguments that forest should be retained within or adjacent to oil palm monocultures for the provision of ecosystem services that benefit yield. We urge for more nuanced assessments of the impacts of forest and biodiversity on yields in crop monocultures to better understand their role in sustainable agriculture.

## Introduction

More than 50% of the global land area that is purportedly suitable for agriculture has already been converted to farmland [1]. Moreover, by 2050, projections suggest that an increase of one billion hectares in agricultural land is required to feed a growing population and to meet increasing consumption per capita [2], much of which will come at the expense of natural habitat in the tropics [3]. Following agricultural development, the landscape is often left with highly fragmented patches of natural habitat that create sharp habitat boundaries with agriculture, and with remaining patches of natural habitat showing varying degrees of degradation and isolation [4], [5]. The simplification of vegetation structure and altered environmental conditions within the agricultural matrix often prove too extreme for much native biodiversity to persist, and valuable ecosystem services may also be threatened by the loss of natural habitats [4], [6]–[8]. Consequently, agricultural expansion is one of the key threats to biodiversity [1], [2], and there is an increasing strain between conserving biodiversity and maximising agricultural production [8]–[10].

Many crops are highly dependent on functional interactions provided by biodiversity, such as soil nutrient supply, pollination, and biological pest control [11]–[14]. Integration of remnant natural habitat features such as forest fragments, riparian strips, and hedgerows within agricultural landscapes is advocated as a means to enhance ecosystem services and thus yield, in addition to providing conservation benefits to native biodiversity, within sustainable landscapes [15]–[21]. While there is a large literature on how the retention of natural habitat can encourage biodiversity and ecosystem services, there is a lack of knowledge of the degree to which remnant habitat might negatively affect yield. The spill-over of biodiversity from natural habitats to agricultural land can negatively alter species diversity and food web interactions [22], [23], with ecosystem dis-services potentially arising as a consequence of providing reservoir populations of insect or fungal pests, crop raiders, invasive weeds, or predators and parasites of beneficial species [12], [23].

Retaining natural habitat remnants within agricultural landscapes also reduces the land available for growing crops, and so may constitute an opportunity cost to local production as well as potentially increasing the demand for converting land elsewhere to agriculture [1]. Landscape-scale planning for agricultural sustainability and conservation therefore hinges on whether or not remnant habitat features provide a net benefit for agricultural production, for conservation, or for both. This is a particularly important issue in the tropics, where conversion to agriculture consumed 1.4% of the tropical forest biome between 2000 and 2005 [24]. To date, research on the relationship between natural vegetation cover and crop yield in the tropics had focused on two agro-forestry crops: coffee [8], [17], [18], [25], [26] and cocao [27], [28], [29]. Both coffee and cocao plantations consist of a mix of crop plants and (non)-native shade trees, which results in an agro-forestry matrix that is comparatively hospitable to forest species (e.g., [30]), and can enhance spill-over from forest and resulting ecosystem services. Consequently, these studies found that close proximity to forest improved pollinator bee numbers [31] and thus coffee yields by up to 20% [18] compared to locations 1,400–1,600 m from forest, and that distance to forest had a marginal positive effect on yield in cacao plantations [27], which have increasing numbers of predatory ant and spider species with higher densities of native shade trees [28]. Furthermore, exclusion experiments showed that bird and bat predation, and the extent of forest cover were important in controlling pest populations and thus positively impacting yield [8], [29].

To our knowledge, the impact of forest on yield has not been assessed in the context of tropical crop monocultures, in which a single crop species is planted in stands that do not contain non-crop trees or other crop species, yet the majority of crop expansion within the tropics now creates monocultures of sugar cane, soya, oil palm, and even cocao. Oil palm *Elaeis guineensis* is one of the world's highest yielding and most financially lucrative monoculture crops [32]. As such, it is expanding very rapidly, with production increasing by >5.5 million ha between 2001 and 2011 [33] and with the majority of this expansion occurring at the expense of hyperdiverse tropical rainforest in Southeast Asia [34]. Unlike coffee and cocao plantations, which can retain high levels of within-plantation biodiversity, whole-sale forest conversion to oil palm results in dramatic local extinctions of most forest-dwelling species [35]–[37]. To reduce the environmental footprint of oil palm, The Roundtable for Sustainable Palm Oil (RSPO), via the high conservation value (HCV) forest protocol [38], [39], and conservation scientists (e.g., [40], [41]) have both highlighted the potential benefits of creating oil palm landscapes that retain forest remnants and riparian strips within plantations, but the net effect of such management on oil palm yield is not known [42].

In this study, we explore the impacts of the local extent of forest cover and the proximity to forest on oil palm yields in Sabah, Malaysian Borneo, where palm oil production covers 19% of the state land area [43] and where there is increasing pressure for further expansion. We thus assess whether the retention of forest within and adjacent to oil palm plantations has a positive, negative or neutral impact on oil palm yield, with the aim of informing sustainable land-use planning.

## Materials and Methods

### Study Area

**Figure 1:**
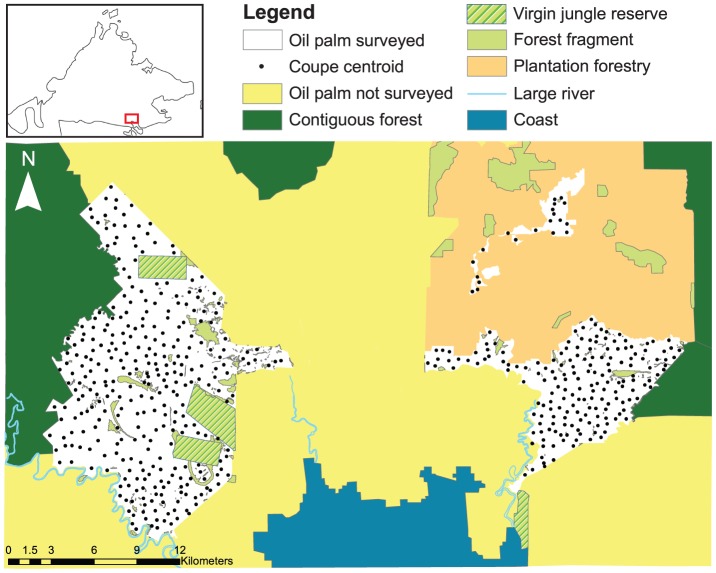
Different land-use types within the study area. The inset shows Sabah, Northeast Borneo, and the red box denotes the study area.

Our study landscape spans 49.5 km×29.8 km (total area = 1474 km^2^ or 147,400 ha) in Sabah, Malaysian Borneo (Figure 1). The landscape comprises >91,000 ha of contiguous oil palm plantations owned by multiple companies, plus a single >28,000 ha block of plantation forestry (*Eucalyptus* spp., Teak, *Acacia* spp.; Sabah Softwoods Bhd.) (Figure 1). All of the soils within our study oil palm plantations are Acrisols, as defined by the World Reference Base for Soil Resourses [44]. However, these soils also contain other main soil components (e.g., Luvisols, Cambisols, etc.) and they have a mixture of alluvium, mudstone, sandstone and igneous rock as parent material [45]; these are combined into ten soil groups (Table S1, Figure S1). Study oil palm plantations also span an elevational range from 10 to 379 m a.s.l. (Figure S2).

Surrounding these plantations are two areas of contiguous lowland dipterocarp forest >100,000 ha in size, which were not bounded by our study area: to the west and north is the Yayasan Sabah (YS) logging concession and to the east is the Ulu Kalumpang forest reserve (itself contiguous with Tawau Hills National Park). Surrounding contiguous forests have both undergone at least two rotations of selective logging [32], [46]. To the south of our study area is a coastline of tidal mangrove creeks, >2 km from the nearest oil palm coupe.

We focus on the oil palm of a single company—Sabah Softwoods Bhd. (we thank Sabah Softwoods Bhd. for providing data, logistical support and site access), a subsidiary of the state-owned Yayasan Sabah Group—with ≈23,000 ha of plantings (Figure 1, in white). Oil palm plantings are separated into three separate zones, which are 2.5 to 9.3 km apart, partitioned by other oil palm plantations between the western and eastern blocks and by plantation forestry between the two eastern blocks (Figure 1). Each zone is sub-divided into discrete parcels known as coupes (n_total_ = 499), which vary in size from 3 to 89 ha (mean±SE: 45±0.7 ha) and which are planted with a density of 100 palms per ha [36].

The Sabah Softwoods oil palm plantations border both contiguous areas of forest, plus numerous isolated forest fragments, increasing in size from tiny patches to large fragments of dipterocarp forest. Forest fragments are divided into Virgin Jungle Reserves (VJRs), which are large (n = 4; mean±SE: 813.95±197.6 ha), were gazetted prior to industrial-scale logging, and thus contain mostly primary forest; whereas privately owned patches (herein ‘private fragments’) tend to be smaller (n = 307, 11.5±4.2 ha, range = 0.01 to 886 ha), to have been selectively logged at least once (the precise logging history of each fragment is unknown) and open to other disturbances (e.g., hunting). Forest fragments were typically retained within plantations due to their steepness and/or unfavourable underlying substrate.

### Oil Palm Yields

Yield data were fresh fruit bunch (FFB) weights (metric tonnes) per hectare for individual coupes from 2008 to 2010. Sabah Softwoods employees visit each oil palm tree within a coupe to harvest ripe fruit bunches and cut decaying fronds twice per month. Bunches are collected into trailors and weighed at the depot. We were provided with the total weight of fruit bunches collected in each coupe on a yearly basis. Oil palm age varied across coupes, from 3 to 15 years old, and because yield varies with age of an oil palm [47] we used the *deviation from the mean expected yield by age* (i.e., observed yield - mean yield for the age of palm) as our indication of yield per coupe. A positive value indicates greater yield than expected, while a negative value indicates a lower yield than expected, given the age of the oil palm. Observed yield data were used from all 499 coupes in 2010. Expected yield was calculated from two yield-by-age curves: firstly, generated from the subset of coupes for which data were provided in 2008 (n = 240 coupes) and 2009 (n = 400; yldSS), and secondly from Butler *et al.* [47] using their average FFB curve (yldB; Figure S3).

### Quantifying Extent of Forest Cover and Proximity to Forest

Forest coverage maps were supplied by Sabah Softwoods, and supplemented with additional maps obtained from the literature [43], [48] and Google Earth images from 2009. The extent of dipterocarp forest cover surrounding and within each oil palm coupe was calculated within circles of radii 100 m, 250 m, 500 m and 1,000 m from the centroid of each coupe. Radii thus span a range of spatial scales relevant to different taxonomic groups, as determined by observations of species' movements between forest and oil palm [49]. From these four radii, an inverse distance-weighted measure of forest-cover area as a proportion of the 1000-m radius circle areaF*_IDW_* was calculated, giving greater weight to forest area closer to a coupe centroid than forest further away [50], [51], using the formula:
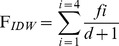
where f*i* is the proportion of forest within a buffer ring (0–100 m, 100–250 m, 250–500 m, and 500–1,000 m) and d (m) is the mean distance of a buffer ring.

Dipterocarp forest included three qualitatively different classes that differed in size and/or logging history, and thus vegetation composition and species communities (e.g., [46], [52], [53], namely (i) contiguous forest, (ii) Virgin Jungle Reserves, and (iii) private fragments. To account for this variation, we also assessed proximity to these dipterocarp forest classes by calculating, from each coupe centroid, the shortest distance to each class. We also calculated distance to plantation forestry, which directly borders some oil palm coupes and which in this study area has more bird biodiversity than local oil palm [54], [55], largely due to the secondary forest understorey that develops under plantation trees. In addition, we included the distance to the nearest surrounding oil palm (i.e. not owned by Sabah Softwoods Bhd.) since a coupe located within a large expanse of oil palm monoculture could benefit if dis-services such as pest infestations originate from within forest or could be disadvantaged if they develop within oil palm. Finally, we evaluated the proximity of the nearest large river from each coupe centroid, the mean elevation across the coupe, and the dominant soil type by area (mean dominant soil coverage was 96.4%±0.01 SE of coupe area), because these environmental variables have the potential to influence oil palm growth and yield. Elevation (m a.s.l.) was calculated from a digital elevation model at 90 m resolution [56]. Soil types were grouped into ten categories (see above; Table S1) and were assessed using a regional soil survey map at 1∶250000 scale [45].

### Statistical Analysis

We used Generalised Least Square models (GLS) to firstly test whether the distance-weighted proportional area of forest affected oil palm yield at the coupe level. The distance-weighted measure of forest cover was square-root transformed to reduce the influence of two outliers. Secondly, we used a GLS to test whether proximity of a coupe centroid to the nearest dipterocarp forest class (contiguous forest; VJR; private fragment) affected oil palm yield. Distance to the nearest forest class was square-root transformed to account for the likely declining effect of forest and the associated reduction of biodiversity spill-over at increasing distances [27]. Additionally, the area of the nearest private fragment was also included as a covariate in proximity models, because different sized fragments could export different levels of services or dis-services. In both cases, the minimum adequate model was achieved by a model selection process comparing nested models [57]. All models included proximity to tree plantation, proximity to large river, proximity to other oil palm plantation, mean elevation and dominant soil type as fixed effects. All models also included a correlation structure using the latitude and longitude of the coupe centroids to account for spatial autocorrelation [58]. Lastly, using our model residuals with 1000 repetitions, we performed a Monte-Carlo permutation test for Moran's I statistic (moran.mc within spdep package) to test whether our results were influenced by spatial autocorrelation (i.e., that the correlation structure had effectively accounted for impacts of space). All spatial analyses were run in ArcGIS 10.0 [59] and all statistical analyses were run in R 2.15.2 [60].

## Results

**Figure 2:**
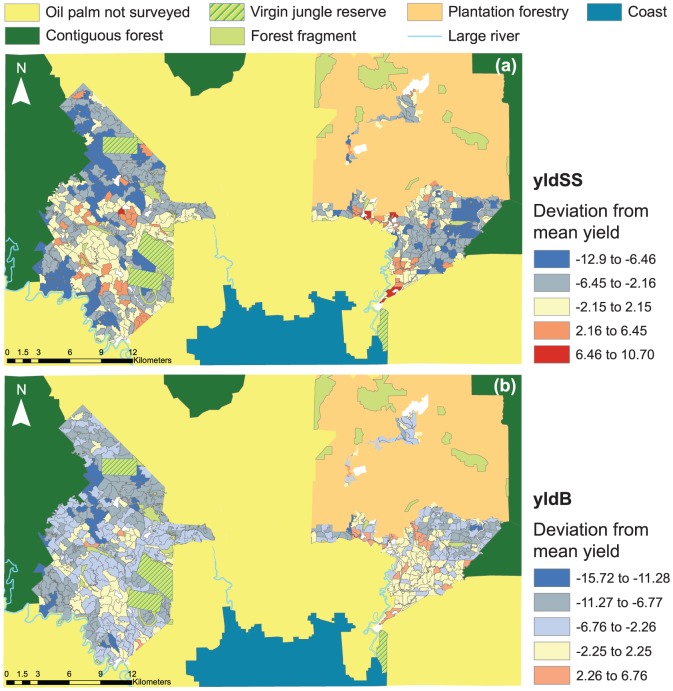
The variation in oil palm yield with adjacent land-uses across the study area. Oil palm yield is measured as the mean deviation from yield-by-age curves (a) generated from the study area data (yldSS), and (b) published by Butler et al. [47] (yldB). Yield is quantified as the fresh fruit bunch weight per hectare (mt ha^−1^).

Oil palm coupes within the landscape spanned a range of distances to forest and degrees of forest cover (Table 1), with the percentage of forest cover at 1000 m ranging from 0 to 79% and distances to forest classes from 30 m and 20.7 km (Table 1), indicating a perfect landscape within which to test the impacts of forest on oil palm yield. Across the study area, there was also a large variation in oil palm yield, spanning over an order of magnitude from 0.12 to 33.46 mt ha^−1^ (Table 1), with a strong correlation between yield and oil palm age (r^2^ = 0.88). However, having accounted for the increase in yield with palm age (see **Materials and Methods**), the spatial distribution of oil palm yield in relation to forest cover showed no clear visual pattern, with a mix of high yield oil palm both close and far from major blocks of forest (Figure 2a, b), and with the same visual pattern for lower yields.

**Table 1:** The range and mean (±SE) of oil palm yield, elevation, and nearest distance to different forest classes, forestry plantations, large rivers and other (not within Sabah Softwoods Bhd.) oil palm plantations within 499 oil palm coupes in Sabah, Malaysian Borneo.

Measure	Maximum	Minimum	Mean	SE
2010 oil palm yield (mt ha^−1^)	33.46	0.12	16.82	0.39
Elevation (m.a.s.l.)	393.53	7.83	127.51	3.11
Forest cover (%) within radii:				
100 m	36.00	0.00	0.18	0.08
250 m	70.00	0.00	1.43	0.24
500 m	83.00	0.00	3.74	0.38
1000 m	79.00	0.00	6.43	0.51
Distance (km) to nearest:				
Contiguous forest	14.63	0.12	5.03	0.15
Virgin forest reserve (VJR)	20.71	0.05	5.93	0.19
Privately owned fragment	3.89	0.03	0.84	0.03
Plantation forestry	26.95	0.09	13.35	0.41
Large river	16.06	0.20	5.79	0.16
Other oil palm	8.66	0.04	2.96	0.10

### Yield Response to Forest Cover

The distance-weighted area of forest cover was retained by the minimum adequate model (MAM), but it was not a significant predictor when yield was derived from either yield-by-age curves: i) the yield-by-age curve generated using Sabah Softwoods coupes (yldSS; GLS: *t*_499_ = 1.52, *P* = 0.13), and ii) Butler *et al.'s* [47] average FFB yield-by-age curve (yldB; *t*_499_ = 1.03, *P* = 0.30) (Table 2). The environmental variables of elevation and distance to nearest forestry plantation were found to be significant predictors when yield was derived from Butler *et al.'s* [47] average FFB yield-by-age curve (yldB; elevation: *t*_499_ = −3.93, *P*<0.01, plantation: *t*_499_ = −3.05, *P*<0.01) (Table 2). All model residuals had no spatial autocorrelation (*P*≥0.39).

**Table 2:** The estimates and parameter coefficients from the minimum adequate generalised least square models testing the effects of forest cover and forest proximity on oil palm yield across the study landscape in Sabah, Malaysian Borneo.

Model	Parameter	Estimate	SE	T	*P*
Forest cover (yldSS*)					
	(Intercept)	−3.1284	0.6915	−4.5240	0.0000
	*forest cover*	20.2703	13.3163	1.5222	0.1286
Forest cover (yldB§)					
	(Intercept)	−0.6051	1.0429	−0.5802	0.5620
	forest cover	13.1835	12.7962	1.0303	0.3034
	**elevation**	**−0.0162**	**0.0041**	**−3.9334**	**0.0001**
	**tree plantation**	**−0.0002**	**0.0001**	**−3.0541**	**0.0024**
Forest proximity (yldSS)					
	(Intercept)	−1.2576	5.7548	−0.2185	0.8271
	contiguous forest	−0.0042	0.0423	−0.0985	0.9216
Forest proximity (yldB)					
	(Intercept)	−0.9506	1.2752	−0.7455	0.4563
	**elevation**	**−0.0143**	**0.0041**	**−3.4587**	**0.0006**
	**tree plantation**	**−0.0002**	**0.0001**	**−2.2356**	**0.0258**

* yldSS – yield estimate derived from the yield-by-age curve generated from Sabah Softwoods coupes.

§ yldB - yield estimate derived from the Butler et al.'s [43] average FFB yield-by-age curve.

Bold indicates significance at *P*<0.001.

### Yield Response to Forest Proximity

**Figure 3:**
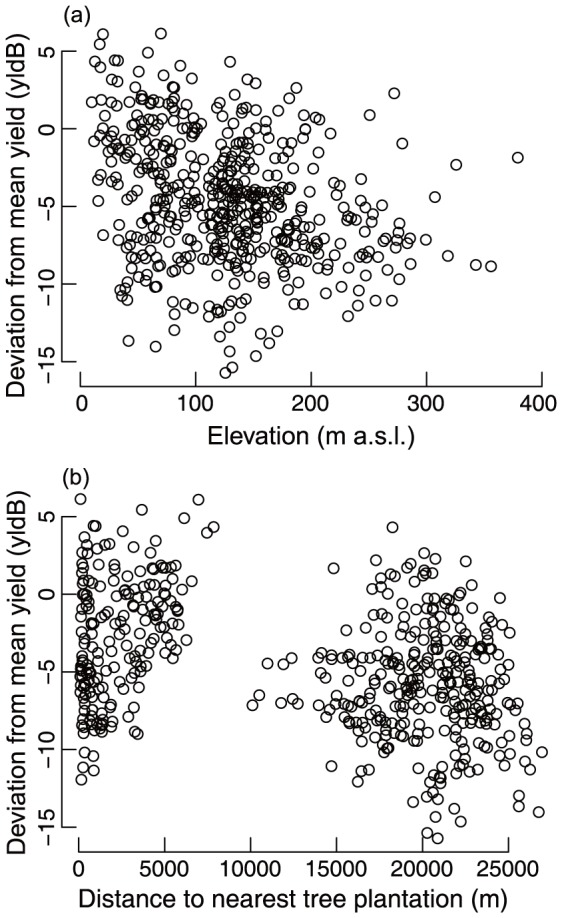
The relationship between oil palm yield and (a) elevation (m a.s.l.), and (b) distance to nearest non-native tree plantation. Oil palm yield was measured as the mean deviation from the yield-by-age curve generated from Butler et al. [47] (yldB), and is quantified as the fresh fruit bunch weight per hectare (mt ha^−1^).

Proximity to any of the three classes of dipterocarp forest (contiguous, VJR, or private fragment) did not have a significant effect on oil palm yield when considering yield derived from either yield-by-age curves (Table 2). Instead environmental variables were more important predictors when oil palm yield was derived from Butler *et al.'s* [47] average FFB yield-by-age curve. Increasing elevation (Figure 3a; *t*_499_ = −3.46, *P*<0.01) and increasing distance from tree plantation (Figure 3b; *t*_499_ = −2.24, *P* = 0.03) both had a significant negative effect on yield (Table 2). Proximity to large river or other oil palm plantation, size of private fragment, and soil type were not significant predictors of yield when using either yield-by-age curve. All model residuals had no spatial autocorrelation (*P*≥0.06).

## Discussion

Agricultural expansion in the tropics is a key driver of the global biodiversity crisis. Pressure to mitigate threats from agriculture and improve sustainability has encouraged suggestions that the retention of natural habitat patches within and adjacent to tropical agriculture would result in the export of ecosystem services [14], [42], [61]–[63], including to oil palm [40], [41]. Yet the potential for spill-over of biodiversity from these features into the agricultural landscape [31], [49], and in turn, whether this impacts upon crop yields positively or negatively has only received attention in the context of coffee and cocao agro-forestry plantations [17], [18], [25]–[28]. Our study is thus the first to focus on the link between forest and crop yield in a tropical monoculture crop, focusing specifically on oil palm, which is rapidly expanding at the expense of forest and highly lucrative. Spill-over from forest is difficult to quantify [64], especially across large scales and when there are various taxa that may spill-over to different degrees and have contrasting impacts. In this study, we instead assess the impacts of the extent of local forest cover and of forest proximity on oil palm yield directly; we therefore did not focus on biodiversity *per se*, and a precise link between biodiversity and yield is absent.

Using both forest cover and proximity metrics, we found that the retention of dipterocarp forest had no significant effect on yield in oil palm monocultures, whereas the environmental variables of elevation and proximity to tree plantations did. These results provide a cautionary note for arguments that forest retention within monoculture landscapes can enhance ecosystem service provisioning and thus improve crop yields [14], [42], [61]–[63]. They also do not support concerns that ecosystem dis-services, such as increased pest populations or mammal crop raiders, are a major issue resulting from the protection of HCV forests under the RSPO. Because we did not directly measure either ecosystem benefits or dis-services, we do not rule out that these are occurring. Rather, our results suggest that either there is an equal balance between ecosystem service benefits and dis-services, resulting in a net neutral impact on yield, or that there is no spill-over occurring. Across our monoculture landscape, it is likely to be a combination of these possibilities, with the former more likely close to forest where species are known to spill-over into oil palm, and the latter more likely far from forest.

Our results suggest that there is no economic rationale for greater forest protection within and adjacent to oil palm monocultures. However, we acknowledge that riparian forest strips and larger fragments may have other important roles. They could provide hydrological and erosion prevention benefits, which might have longer-term benefits that cannot be quantified by focusing only on a single year of oil palm yield. These features could also provide biological benefits, harbouring some biodiversity [36], [52], [53] or by acting as stepping-stones and corridors for dispersal of species through the oil palm matrix [19], [49], which could be vital for retaining meta-population dynamics.

The optimum growing conditions of oil palm (*Elaeis sp.*) are in lowland wet tropics of <1000 m elevation [65]: the negative effect of increasing elevation on yield is thus not surprising. This result highlights the limitation for future expansion of oil palm, especially in regions such as Southeast Asia where many of the prime locations have already been developed, and less optimum areas are already being considered and converted for oil palm development [66]. Proximity to tree plantations may provide some positive spillover, for example pest predation by birds, which are supported in greater numbers in tree plantations than oil palm [54], [55]. In other agricultural systems multi-cropping has been found to be beneficial ([14] and references there in, [67]), and this is an important future direction for optimal agricultural landscape design. However, these results should be interpreted with caution, because elevation and proximity to tree plantation are positively correlated (Pearson's correlation: r = 0.12, p = 0.02), with lower lying areas of higher oil palm yield also closer on average to tree plantations.

In this study, we did not consider the potential impacts of different management activities, such as the use of pesticides or permitting the growth of understory vegetation, or of palm condition (e.g. pest abundance, disease, or structural damage) on yield, which represent important next steps to disentangle drivers of yield change [42]. With the exception of VJRs, which have only been lightly logged in patches, all of the forests in the study area have been selectively logged on an intensive, industrial scale. It is plausible that proximity to primary, unlogged forest could impact differently upon yield. However, this seems unlikely because previous work in the region has shown the retention of high levels of biodiversity, including most primary forest species [46], [68], [69], and high functional diversity [70], [71] within contiguous blocks of logged forests. It is also possible that ecological services or dis-services from forest could affect palm oil quality, and hence price. Finally, we only focused on Southeast Asia and on one monoculture crop, and there could be different relationships between forest and yield in other tropical biomes, where oil palm is now expanding rapidly [72], or with other crops such as soya and sugar cane.

### Conclusion

Our results show a neutral effect of forest on oil palm yield. Consequently, dipterocarp forests appear neither to export sufficient ecosystem service benefits to result in a net increase in yield nor to export sufficient ecosystem dis-services to result in a net reduction of yield within oil palm plantations. We thus observe no evidence to support arguments for the retention of forest for the provision of ecosystem services explicitly for yield benefits within oil palm monocultures [42], [62]. Many arguments have been made for implementing an integrated framework of agricultural design, which considers biodiversity conservation, ecosystem services and agricultural output [42], [73], [74]. These are to be warmly welcomed, but in light of our study the proposed benefits of such designer landscapes within monocultures should avoid couching arguments for forest retention in the context of yield benefits. We finish by urging for more empirical assessments of the impacts of forest and biodiversity on crop monoculture yields to better understand their potential role in sustainable agriculture: we fear that by resting arguments for the retention of forest on improved oil palm yield, there could be unintended consequences such as the clearance of retained forest patches and thus the removal of refugia for biodiversity if no such empirical support were to emerge.

## Supporting Information

File S1 **Table S1. Description of soil types found across the study area. Figure S1. Distribution of soil types across the study area. Figure S2. Distribution of elevation across the study area. Figure S3. Oil palm yield-by-age curves.**
(DOCX)
