# Supplementary material for: Sustainable Management in Crop Monocultures: The Impact of Retaining Forest on Oil Palm Yield
Source: PLoS One. 2014 Mar 17;9(3):e91695. doi: 10.1371/journal.pone.0091695 (PMC3956724; doi:10.1371/journal.pone.0091695)
Supplement: File S1 — Table S1. Description of soil types found across the study area. Figure S1. Distribution of soil types across the study area. Figure S2. Distribution of elevation across the study area. Figure S3. Oil palm yield-by-age curves. (DOCX) [file pone.0091695.s001.docx]

**Supporting Information**

**Sustainable Management in Crop Monocultures: The Impact of Retaining Forest on Oil Palm Yield**

**Felicity A. Edwards^1*^, David P. Edwards^2,3^, Sean Sloan^2^, Keith C. Hamer^1^**

**1** School of Biology, University of Leeds, Leeds, LS2 9JT, United Kingdom, **2** Centre for Tropical Environmental and Sustainability Science (TESS) and School of Marine and Tropical Biology, James Cook University, Cairns, Queensland 4878, Australia, **3** Department of Animal and Plant Sciences, University of Sheffield, Sheffield, S10 2TN, United Kingdom

* E-mail: [bs08f2a@leeds.ac.uk](mailto:bs08f2a@leeds.ac.uk)

**Contents:**

Table S1: Description of soil types found across the study area.

Figure S1: Distribution of soil types across the study area.

Figure S2: Distribution of elevation across the study area.

Figure S3: Oil palm yield-by-age curves.

**Table S1:** The description of soil types across the study area in Sabah, Malaysian Borneo. The Soil association refers to the soil name from The Soils of Sabah map [1], Parent material describes the underlying geology, and the Main soil units and Sub soil units refer to the individual characteristics of the soil. Asterisks refer to those soils grouped for analyses.

| **Soil association** | **Parent material** | **Main soil untis** | **Sub soil units** |
| --- | --- | --- | --- |
| Kinabatangan* | Alluvium | Acrisol | Gleyic |
|  |  | Luvisol | Gleyic |
|  |  | Gleysol | Dystric, Eutric and Humic |
| Labau* | Alluvium | Acrisol | Orthic |
|  |  | Cambisol | Dystric and Gleyic |
|  |  | Fluvisol | Dystric and Eutric |
| Brantian* | Alluvium | Acrisol | Ferric, Gleyic, and Orthic |
|  |  | Podzol | Gleyic |
| Kalabakan | Mudstone and Sandstone | Acrisol | Ferric and Orthic |
|  |  | Luvisol | Chromic, Ferric, and Orthic |
| Mawing | Mudstone and Sandstone | Acrisol | Orthic |
|  |  | Cambisol | Dystric |
| Dalit | Mudstone, Sandstone, Alluvium | Acrisol | Ferric, Gleyic, and Orthic |
| Kretam | Mudstone, Sandstone, and Miscellaneous rocks | Acrisol | Orthic and Ferric |
|  |  | Luvisol | Ferric, Chromic and Orthic |
| Lokan | Mudstone, Sandstone, Alluvium | Acrisol | Orthic |
|  |  | Cambisol | Dystric |
| Bang | Mudstone, Sandstone, and Miscellaneous rocks | Acrisol | Orthic |
|  |  | Cambisol | Dystric |
| Gumpai | Mudstone, Sandstone, and Miscellaneous rocks | Acrisol | Orthic |
|  |  | Luvisol | Orthic |
|  |  | Cambisol | Dystric and Eutric |
|  |  | Lithosol |  |
| Crocker | Mudstone and Sandstone | Acrisol | Orthic |
|  |  | Cambisol | Chromic and Dystric |
|  |  | Lithosol |  |
| Maliau | Mudstone and Sandstone | Acrisol | Orthic |
|  |  | Cambisol | Dystric |
|  |  | Gleysol | Humic |
|  |  | Podzol | Gleyic |
|  |  | Lithosol |  |

**
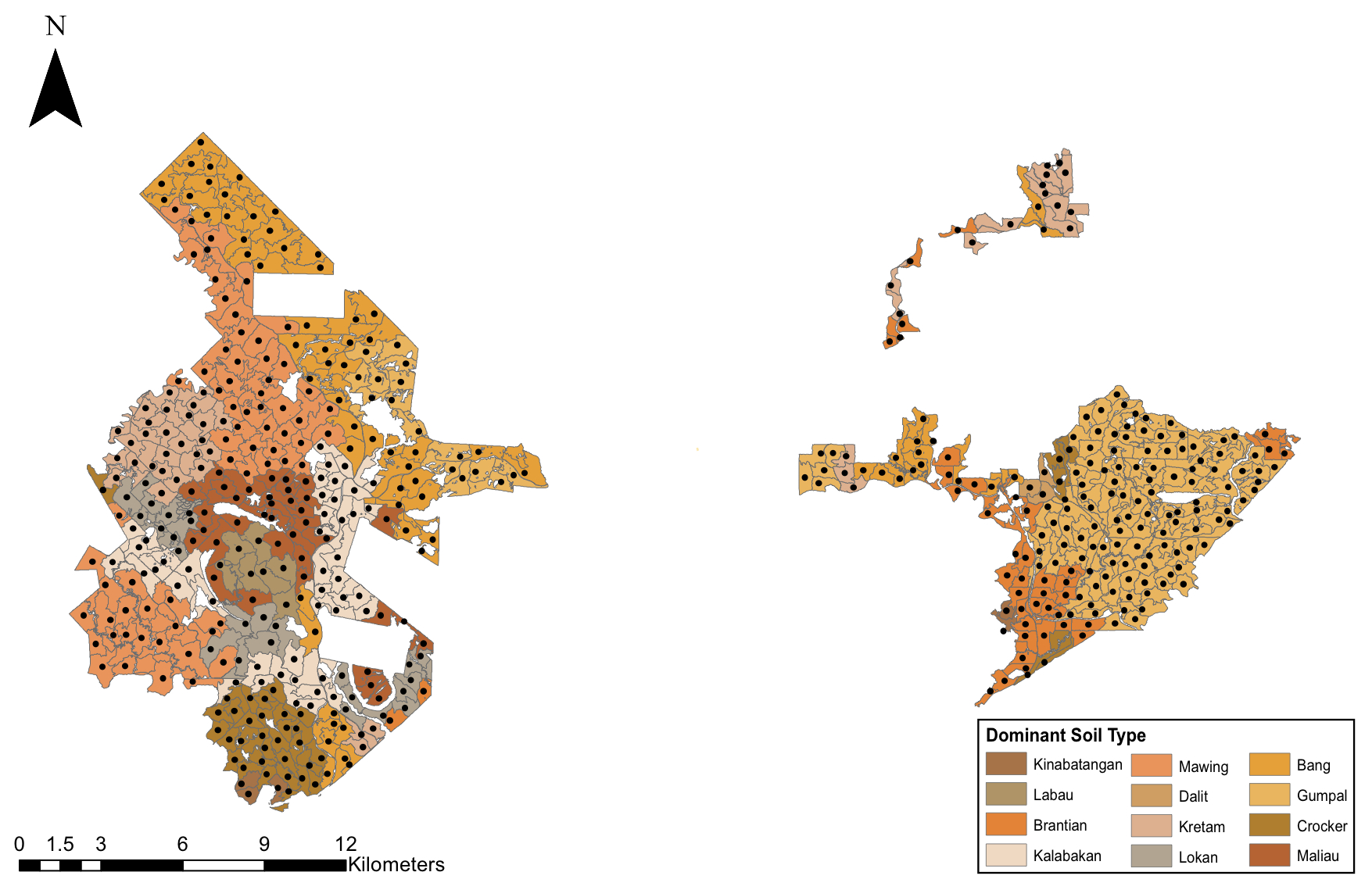
Figure S1:**

**Figure S1: The distribution of the dominant soil type per oil palm coupe across the study area in Sabah, Malaysian Borneo.** Soil types are classified from The Soils of Sabah Map [1] and refer to the dominant soil type by area.

**
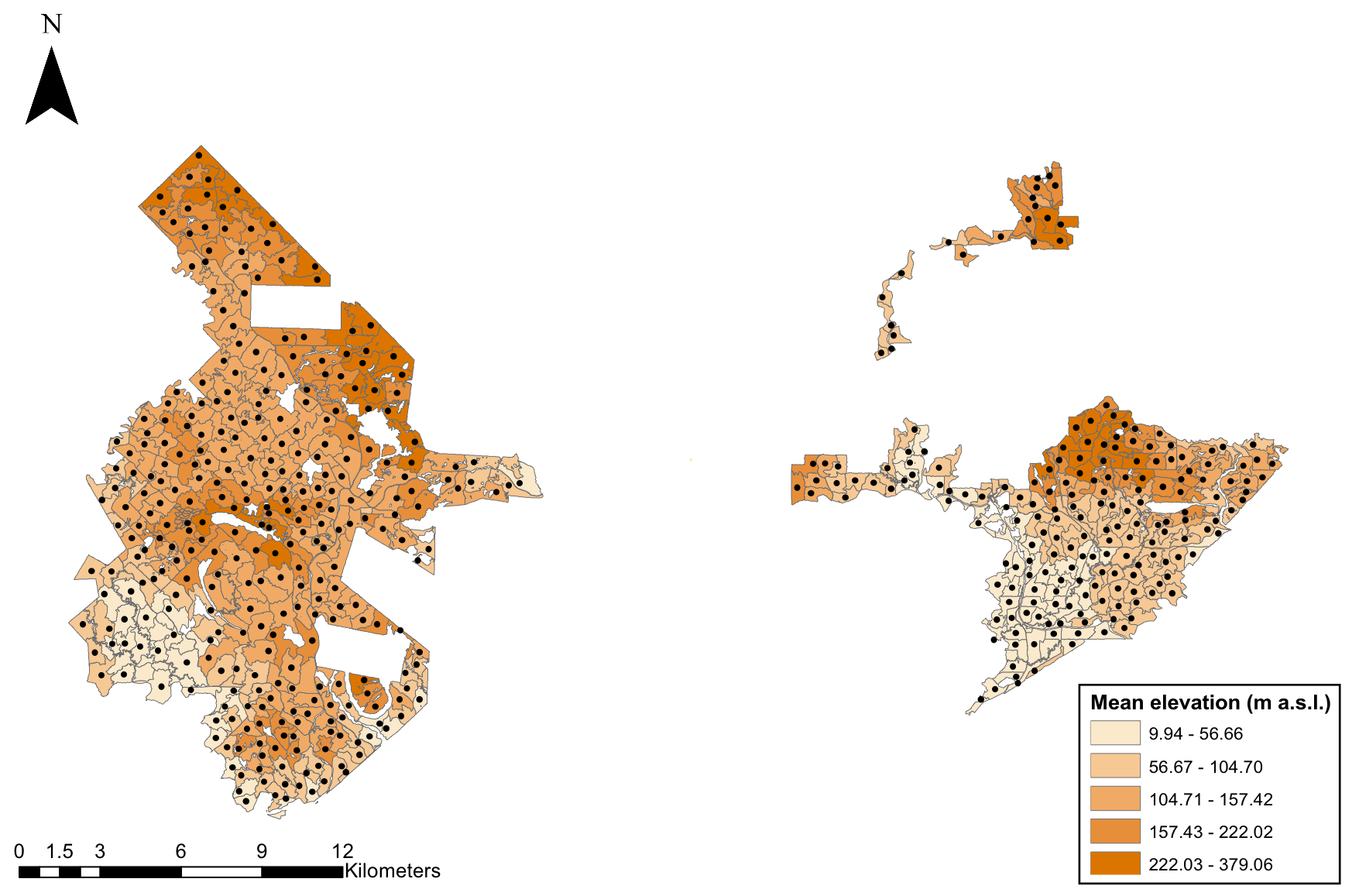
Figure S2:**

**Figure S2: The distribution of the mean elevation (m a.s.l.) per oil palm coupe across the study area in Sabah, Malaysian Borneo.**

**Figure S3:**


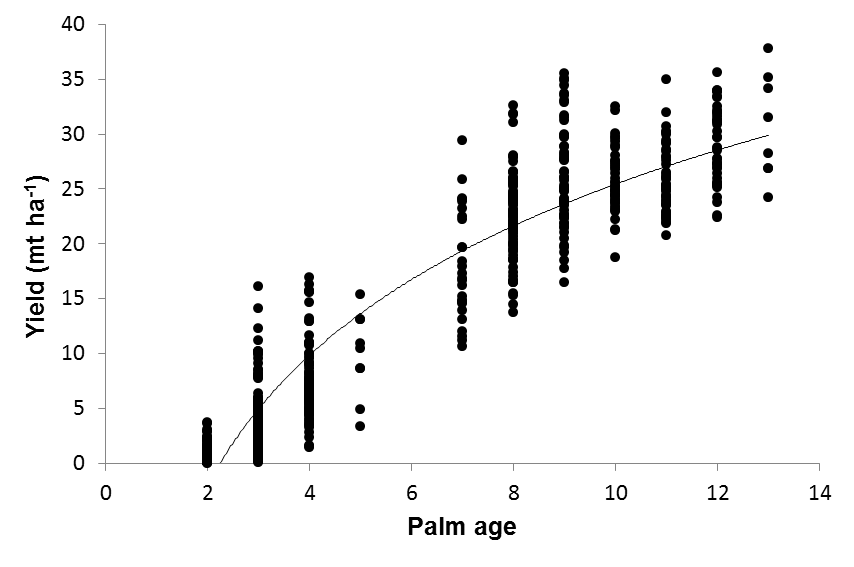

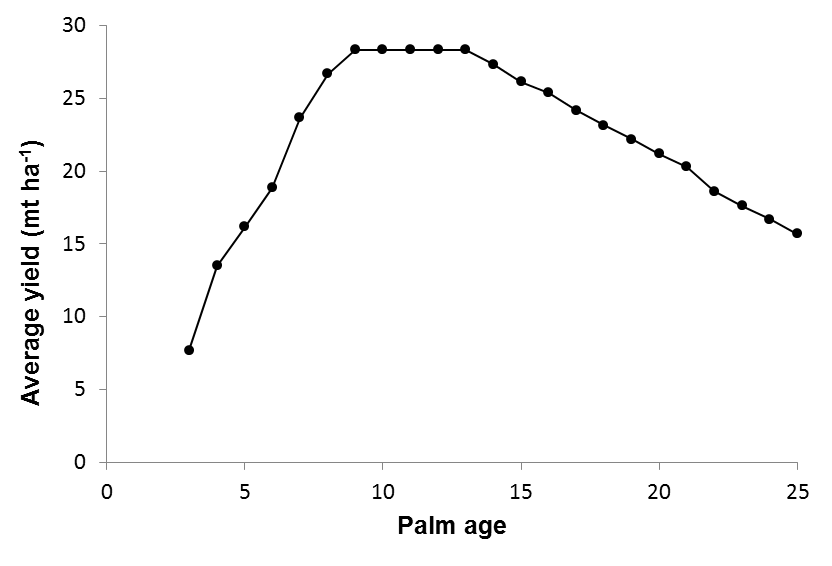


**(a)**

**(b)**

**Figure S3:** Oil palm yield-by-age curves used to calculate the deviation from the mean oil palm yield values. Data was generated from (a) Sabah Softwood plantation for oil palm coupes with available data for 2008 and 2009, and (b) the average yield curve as produced by Butler *et al.* [2]. Oil palm yield is measured as the fresh fruit bunch weight per hectare (mt ha^-1^).

**References**

**[1]** Director of National Mapping (1974) Tawau. The Soils of Sabah. Sheet NB 50-15. D.O.S. 3180J. The British Government's Overseas Development Administration U.K

**[2]** Butler RA, Koh LP, Ghazoul J (2009) REDD in the red: palm oil could undermine carbon payment schemes. Conserv Lett 2: 67-73.
